# Measurement of carrier lifetime in micron-scaled materials using resonant microwave circuits

**DOI:** 10.1038/s41467-019-09602-2

**Published:** 2019-04-09

**Authors:** Sukrith Dev, Yinan Wang, Kyounghwan Kim, Marziyeh Zamiri, Clark Kadlec, Michael Goldflam, Samuel Hawkins, Eric Shaner, Jin Kim, Sanjay Krishna, Monica Allen, Jeffery Allen, Emanuel Tutuc, Daniel Wasserman

**Affiliations:** 1 Electrical and Computer Engineering, Microelectronics Research Center, University of Texas at Austin, Austin, Texas 78758 USA; 2 Materials Science and Engineering, University of Wisconsin, Madison, Wisconsin 53706 USA; 3 Sandia National Laboratories, Albuquerque, NM 87185 USA; 4 Electrical and Computer Engineering, Ohio State University, Columbus, Ohio 43210 USA; 5 Air Force Research Laboratory, Munitions Directorate, Eglin Air Force Base, Eglin, Florida 32542 USA

## Abstract

The measurement of minority carrier lifetimes is vital to determining the material quality and operational bandwidth of a broad range of optoelectronic devices. Typically, these measurements are made by recording the temporal decay of a carrier-concentration-dependent material property following pulsed optical excitation. Such approaches require some combination of efficient emission from the material under test, specialized collection optics, large sample areas, spatially uniform excitation, and/or the fabrication of ohmic contacts, depending on the technique used. In contrast, here we introduce a technique that provides electrical readout of minority carrier lifetimes using a passive microwave resonator circuit. We demonstrate >10^5^ improvement in sensitivity, compared with traditional photoemission decay experiments and the ability to measure carrier dynamics in micron-scale volumes, much smaller than is possible with other techniques. The approach presented is applicable to a wide range of 2D, micro-, or nano-scaled materials, as well as weak emitters or non-radiative materials.

## Abstract (web summary)

A method for measuring carrier dynamics in micron-scale optoelectronic materials based on time-resolved microwave reflection is reported. Compared to a standard time-resolved photoluminescence approach, the authors show a 10^5^ improvement in sensitivity when measuring lifetimes in a semiconductor pixel.

## Introduction

Traditionally, direct electrical measurements of minority carrier lifetimes are made using direct current (DC) photoconductive decay (PCD) measurements, whereas non-invasive, contact-free lifetime measurements are made using time-resolved microwave reflectance (TMR), or time-resolved photoluminescence (TRPL). In TRPL, a short laser pulse optically excites a light-emitting material and the resulting photoluminescence (PL) is then collected as a function of time; carrier lifetimes are then extracted from the temporal decay of the emitted PL^1^. However, in addition to the pulsed laser, TRPL also requires collection optics and a detector that have both been tailored to the wavelength of light emitted by the sample. Hence, oftentimes entirely different optics and photodetectors are required for samples emitting in different regions of the electromagnetic spectrum. In addition, TRPL often requires trade-offs in the choice of detectors, balancing detector sensitivity (allowing for measurement of weakly emitting or small samples) and detector response times (allowing for measurement of shorter carrier lifetimes), with more sensitive detection almost always accompanied by poorer time resolution.

Alternatively, small samples or weak emitters can be measured using the PCD^2,3^ technique, which records the temporal dependence of a sample’s DC conductivity following pulsed excitation. However, such an approach requires fabrication of ohmic contacts to the material under test and thus increased time and expense associated with the contact patterning and metal deposition. The processes for contact formation can vary depending on the material of interest and may not be feasible for materials or structures such as polymers, organic dyes, nanowires, or micron- or nano-scale two-dimensional (2D) materials.

The non-invasive, contact-free analog to PCD is TMR, which records the time evolution of the microwave reflection from a sample following optical excitation, essentially probing a change in the sample’s radio frequency (RF) conductivity^4,5^. Compared with TRPL, TMR has the advantage of improved sensitivity; furthermore, as it is the photo-conductivity that is probed, the sample does not need to emit and there is no need for wavelength-tailored collection optics or a high-speed optical detector. The free-space nature of conventional TMR results in sensitivity to carrier concentrations over large areas (up to ~1 cm^2^), which can complicate analysis or require large diameter optical pump beams when uniform carrier density profiles are desired, as is the case when extracting Auger or radiative coefficients of a material^6^. For micron-scaled samples, the mismatch between the large RF probe and sample area will significantly decrease the TMR signal-to-noise ratio (SNR), as only a small fraction of the reflected RF signal is modulated by the change in the micro-scale sample’s carrier concentration. Similar difficulties would be encountered for TMR measurements of photoexcited carriers in 2D materials, as exfoliation processes typically result in approximately micron-scale flakes of 2D materials^7^.

The interaction of RF signals with optoelectronic materials can be scaled and concentrated to smaller volumes by using a microstrip split-ring resonator (SRR), which, on resonance, provides strong spatial localization of the RF field^8,9^. Comparable structures have been employed in previous RF SRR-sensing demonstrations, which measure changes in the resonator’s RF response, although such an approach was used to investigate large-area patterns and on relatively slow (~s) timescales in the ultraviolet^10,11^.

**Fig. 1:**
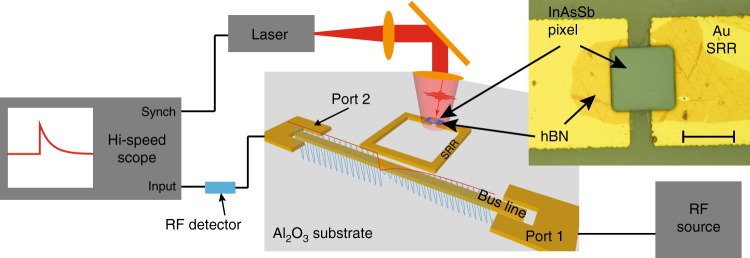
Overview of the proposed technique. A radio frequency (RF) source outputs a continuous wave (CW) microwave signal at the resonant frequency of the split-ring resonator (SRR) through port 1. A pulsed laser excites electron-hole pairs (EHPs) in the material under study (an indium arsenide antimonide pixel in this case) loaded within the split gap of the resonator. The EHPs modulate the CW signal on the microstrip busline, whose envelope function is detected by a Schottky diode RF detector. The modulated signal is then sent to a high-speed oscilloscope synchronized to the laser repetition rate. A micrograph shows both the pixel and a thin layer of insulating hexagonal boron nitride (hBN) loaded into the SRR; the scale bar in the image is 20 μm

Here we present a technique for measuring nanosecond timescale carrier dynamics in small volumes of optoelectronic materials: micro-scale time-resolved microwave resonator response (µ-TRMRR), shown schematically in Fig. 1. Micron-scale infrared (IR) pixel elements of a semiconductor material under test are placed in the split gap of an SRR coupled to a microstrip busline, and when photoexcited, alter the RF circuit’s *S*_21_ parameter (the ratio of the forward traveling voltage waves at port 1 and port 2). Measuring the time evolution of the resonator’s *S*_21_ parameter, at resonance, allows for electrical readout of carrier dynamics in the sample. By using small Ku band (12–18 GHz) resonators and driving the circuit at a single resonant frequency, we are able to effectively characterize high-speed (~ns) carrier dynamics in micron-scale mid-IR materials, something not practically achievable using traditional contact-free lifetime measurement techniques or investigated in previous RF resonator measurements. Specifically, we measure the photoexcited carrier lifetimes in 24 µm × 24 µm pixels of the narrow bandgap III–V material InAsSb, which is of practical interest for mid-wave IR (MWIR) detector applications^12–15^. The same material is measured using TRPL as well as large-area TMR measurements, and we compare the extracted lifetimes from the µ-TRMRR, TMR, and TRPL approaches. We demonstrate a five order of magnitude improvement in the measurement sensitivity compared with TRPL and the ability to extract carrier lifetimes with photoexcitation as weak as 35 fJ incident excitation pulse energy upon the pixel at room temperature. The presented technique offers an extremely sensitive and high-speed (~ns) approach for measuring carrier lifetimes in small volumes of any number of optoelectronic materials.

## Results

### Optical characterization

**Fig. 2:**
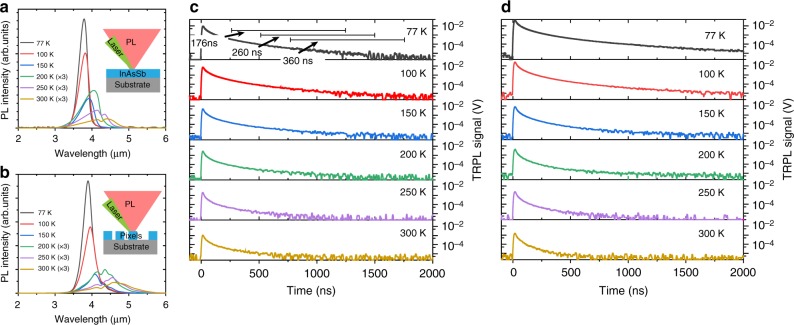
Optical characterization of the material. Temperature dependence of the photoluminescence (PL) spectra of **a** bulk, as-grown InAsSb and **b** a cluster of 24 µm × 24 μm, 1 μm-thick InAsSb pixels placed on thermal release tape. The PL spectra for temperatures greater than 200 K are scaled by a factor of 3 for clarity. Time-resolved photoluminescence (TRPL) data (50,000 averages) from **c** bulk, as-grown InAsSb and **d** 24 µm × 24 μm, 1 μm-thick cluster of InAsSb pixels placed on thermal release tape. The 77 K data in **c** includes lifetimes extracted from shifted fitting intervals; the significant variation in extracted lifetimes for these three intervals points to a deficiency of the TRPL method. For emitters with weak emission at low injection, the TRPL signal to noise is often not sufficient to extract an accurate minority carrier lifetime

Figure 2 shows the temperature-dependent PL and TRPL from bulk, as-grown InAsSb and an array of InAsSb pixels transferred to thermal release tape. The TRPL is measured using a fast HgCdTe (mercury cadmium telluride, MCT) detector, as described in the Methods section. Strong MWIR PL is observed from both the as-grown and pixel array of the narrow-bandgap InAsSb, particularly at low temperature; furthermore, as the temperature increases, the PL intensity decreases (from increased non-radiative effects) and red shifts (from a decrease in the InAsSb bandgap). TRPL measurements on the bulk material (Fig. 2c) give low-injection minority carrier lifetimes varying from 360 to 194 ns as temperature increases from 77 to 300 K. Figure 2d shows the TRPL from the large-area pixel array on thermal release tape. The pixel TRPL lifetime measurement shows a similar temperature dependence as the bulk InAsSb sample, with lifetimes ranging from 381 to 112 ns with increasing temperature. The pixel TRPL signal (Fig. 2d) can only be observed from a large-area array of pixels, as the emission from a single pixel is too weak to be time resolved.

The benefits of the MCT detector used for these TRPL measurements include response out to wavelengths of ~12 μm and a time constant of ~4 ns. However, the faster response time of the MCT comes with a significant sensitivity penalty, making detection of weak emission challenging. As demonstrated in the 77 K data of Fig. 2c, the choice of fitting interval strongly determines the lifetime extracted from TRPL. The low-injection condition (*δn* = *δp* < *n*_0_), where the photoexcited excess carrier concentrations (*δn* = *δp*) are less than the background doping (*n*_0_), is the regime of greatest interest for detector material characterization, as it defines a region of linear detector operation corresponding to typical photon fluxes for IR detection. In low injection, a single exponential fit can be used to extract a minority carrier lifetime. However, weak emitters and/or inefficient detection can result in the TRPL signal falling beneath the system noise floor before the low-injection condition is satisfied. In such a situation, fitting to different portions of the decay signal will give very different lifetimes, as shown in Fig. 2c, making the extraction of an accurate minority carrier lifetime challenging in the case of large-area samples and effectively impossible for a single pixel. This limitation becomes particularly problematic for long wavelength materials, where high-speed and high-sensitivity detectors are harder to come by and where non-radiative recombination can dominate carrier dynamics and make for inefficient emission, especially at non-cryogenic temperatures^16,17^.

### Single-pixel time-resolved microwave resonator response

**Fig. 3:**
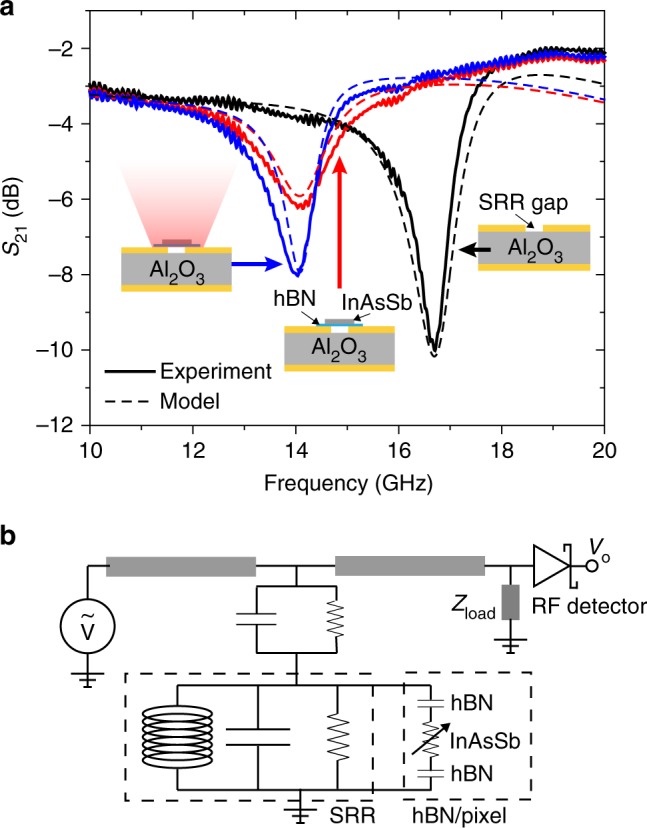
Modeling the coupled pixel-resonator circuit. **a** Experimental (solid) and modeled (dashed) *S*_21_ spectra for microstrip resonator without pixel (black), after loading with hexagonal boron nitride (hBN) and InAsSb pixel (red), and under illumination of the InAsSb pixel post-loading (blue). **b** Equivalent circuit model of a split-ring resonator (SRR) capacitively coupled to a microstrip transmission line terminated by a load. The hBN/pixel structure is depicted as a shunt with parallel capacitance (hBN) and variable conductance (InAsSb pixel)

Although TMR alleviates many of the challenges associated with TRPL, the large free-space microwave probe requires a correspondingly large and uniform optical pump signal. In contrast, the μ-TRMRR approach uses a resonant microwave circuit, which can strongly localize and enhance the RF field, to provide a strong overlap with micro-scale volumes of the material under test. In addition, the use of on-chip microwave probes allows for direct electrical readout of carrier dynamics in our materials. Figure 3a shows the experimental (solid) and modeled (dashed) *S*_21_ parameters for the resonant microwave circuit used in our µ-TRMRR set-up, which consists of a microstrip SRR coupled to a microstrip busline. As the bare pixel is unintentionally doped (UID; see Methods), its dark conductivity can inadvertently short the resonator; furthermore, filling up the air gap with a larger index material capacitively loads the SRR, which can also weaken the resonance. To minimize these effects, a thin layer of insulating hexagonal boron nitride (hBN) is placed before the placement of the pixel, which reduces both shorting and capacitive loading. A clear bandstop feature is observed at ~16.5 GHz for the bare SRR, which shifts to lower frequency (and weakens in magnitude) when the SRR is loaded with the InAsSb pixel and hBN spacer. The transmission line model (described in Methods) for the bare and loaded circuit is shown in Fig. 3b, where the hBN and pixel are modeled as a shunt across the SRR consisting of a series combination of capacitance (hBN) and conductance (pixel). Optical excitation of the pixel generates electron-hole pairs (EHPs), increasing the pixel conductivity and thus modulating the circuit’s *S*_21_ parameter. This modulation of the *S*_21_ parameter can clearly be observed in the modeled and experimental RF spectra of our device under illumination in Fig. 3a, where a significant change in transmitted RF signal can be obtained by changing the conductivity of the micron-scale pixel over the SRR gap via illumination.

**Fig. 4:**
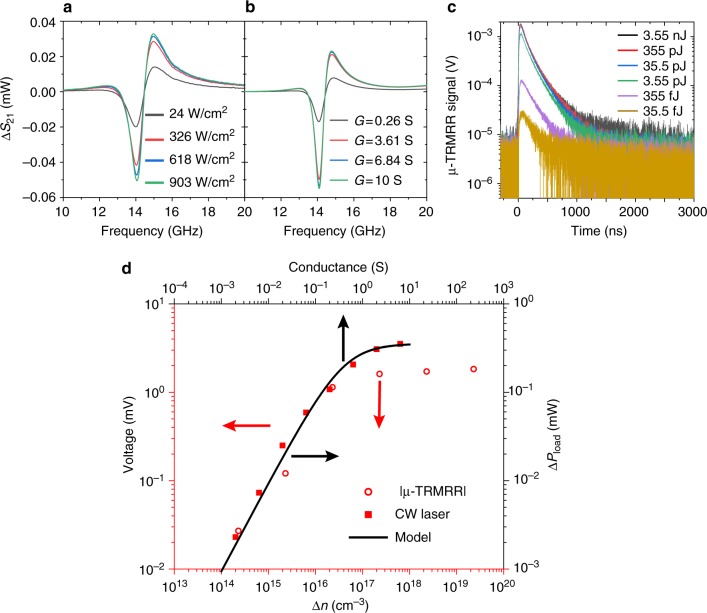
Linearity of the presented technique. **a** Measured Δ*S*_21_ (the change in the *S*_21_ parameter when light is incident upon the pixel) for varying CW laser excitation at room temperature, **b** modeled Δ*S*_21_ as a function of pixel conductance, and **c** measured room-temperature µ-TRMRR signal as a function of energy incident upon the pixel. **d** |Δ*S*_21_| on resonance from CW response shown in **a**, (red squares), and peak |µ-TRMRR| signal shown in **c** (red circles) as a function of excess carrier concentration and modeled | Δ*S*_21_| (black, solid) as a function of conductance, from **b**

Linearity of the measured change in transmitted RF signal $\left( {\Delta S_{21}} \right)$ with respect to carrier concentration is key to extracting accurate, low-injection lifetimes. From the *S*_21_ plots in Fig. 3a, we observe that photoexcitation of carriers results in a negative $\Delta S_{21}$ (when measured on resonance, at the dip in the dark $S_{21}$ spectra), which must limit the dynamic range of the circuit response to the dark *S*_21_ value. Under continuous wave (CW) excitation (Fig. 4a), we see a clear saturation in the $\Delta S_{21}$ with increasing laser intensity, which could result from the circuit response saturation or, alternatively, excitation power-dependent mobility or lifetimes in the InAsSb, the latter coming from the increased contribution of fast non-radiative recombination mechanisms (Auger recombination in particular) to the total recombination rate^6,15,18–20^. However, our modeled $\Delta S_{21}$ (Fig. 4b), as a function of increasing shunt conductance in the circuit, matches the experimental $\Delta S_{21}$ data under CW illumination, suggesting the observed saturation (at these excitation powers) is mostly intrinsic to the SRR circuit and unrelated to carrier lifetime.

Figure 4c shows the excitation pulse energy dependence of the µ-TRMRR signal. Measuring the initial response amplitude |µ-TRMRR| vs. pulse energy, we can obtain an experimental picture of the circuit response (independent of carrier lifetime), which also shows a clear saturation with increasing excitation energy. In Fig. 4d, we plot both the |µ-TRMRR| signal and the circuit response to CW excitation, assuming constant minority carrier lifetime (red axes) as a function of excess carrier concentration (see Methods for details) as well as the modeled circuit response as a function of photo-induced conductance (black axes). Both the |µ-TRMRR| data and CW response show a clear saturation at carrier concentrations greater than ~10^17^ cm^−3^, near or slightly larger than the measured background carrier concentration in the InAsSb. For carrier concentrations less than ~10^16^ cm^−3^, a region corresponding to the low-injection regime, our circuit response remains linear, in agreement with our modeled response for small photo-induced conductances. The results summarized in Fig. 4d suggest that although the circuit response saturates at large excess carrier concentrations, the strong sensitivity to carrier concentration and the linear response of our system across several orders of magnitude of carrier concentration offers a range of operation well-suited for exploring Auger, radiative, and Shockley–Read–Hall lifetimes in IR detector materials. This result points to the primary advantage of our µ-TRMRR technique when compared with TRPL, which requires significant trade-offs between detector sensitivity and speed, and which for fast detector response times and weakly emitting samples, cannot accurately measure carrier dynamics at low carrier concentrations.

### Comparison of lifetime techniques

**Fig. 5:**
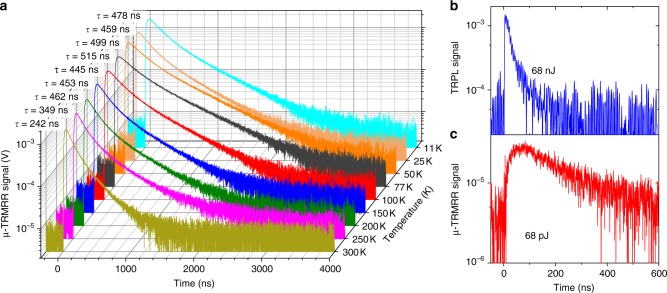
Transient decay curves. **a** Temperature-dependent micro-scale time-resolved microwave resonator response (μ-TRMRR) signal from InAsSb pixel (35.5 pJ pulse energy on pixel, 2000 averages on oscilloscope). Extracted lifetimes are shown above each curve. **b** Time-resolved photoluminescence (TRPL) response of pixel array with 68 nJ laser pulse energy (16.6 nJ incident on pixels) and **c** µ-TRMRR data for a single pixel with 68 pJ pulse energy (35.5 fJ incident on pixel). Both experiments in **b** and **c** are performed at 300 K, with 2000 averages on the oscilloscope. We see that a comparable response is obtained from the μ-TRMRR with >10^5^ less energy than is needed for TRPL

Figure 5a shows the temperature-dependent µ-TRMRR data from the single InAsSb pixel, at the same pump energy used for the TRPL data in Fig. 2 (but with only 2000 averages, compared with the 50,000 averages required for the TRPL). A clear improvement is observed in the µ-TRMRR SNR, with fitting possible to much longer times. Figure 5 also shows a direct comparison of the TRPL data from the array of InAsSb pixels (Fig. 5b) and our µ-TRMRR results from a single InAsSb pixel (Fig. 5c) at 300 K (both with 2000 averages, for an accurate comparison). To achieve SNR adequate to extract a lifetime, the pixel array requires pumping with a pulse energy of 68 nJ, whereas a comparable signal is observed from the µ-TRMRR with only 68 pJ of pulse energy (equivalent to 35.5 fJ incident on the pixel). Even with the three orders of magnitude attenuation in pump energy, the µ-TRMRR allows for fitting to the tail of the emission signal, where the tail of the TRPL emission is well below the noise floor. Thus, the µ-TRMRR approach has significant benefits over TRPL, where weak signals from poor emitters or long carrier lifetimes (effectively stretching photon emission over longer time intervals), can lead to low SNR and thus inaccurate measurement of lifetimes. Moreover, the significant improvement in our time-response data using the µ-TRMRR approach comes from the response of a single pixel, as opposed to the TRPL data, which comes from the collective response of more than 1000 pixels. As TRPL requires three orders of magnitude more power with >1,000 pixels, our µ-TRMRR approach offers at least a ~10^5^ improvement in sensitivity when compared with TRPL performed with a conventional, high-speed MCT. Alternatively, by calculating the absorbed optical energy (see Methods) required to achieve comparable responses from the pixel array (16.6 nJ) and the single pixel in our μ-TRMRR system (35.5 fJ), we similarly obtain a > 10^5^ improvement in sensitivity.

**Fig. 6:**
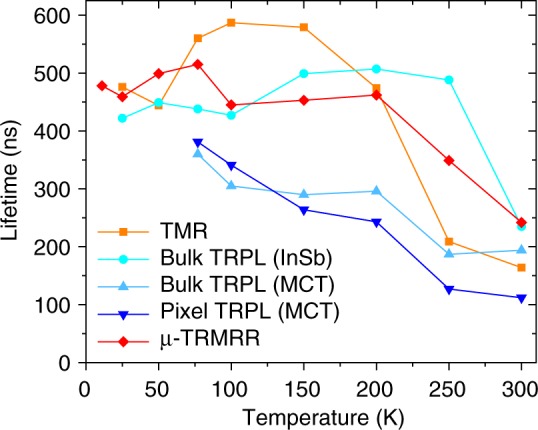
Comparison of lifetime measurement techniques. Extracted carrier lifetimes as a function of temperature for time-resolved microwave reflectance (TMR) on as-grown InAsSb (orange), time-resolved photoluminescence (TRPL) on as-grown InAsSb using InSb (cyan) and mercury cadmium telluride (MCT) (light blue) detectors, TRPL from pixel array using MCT detector (blue), and single-pixel µ-TRMRR (red) techniques. TRPL (InSb), TMR, and μ-TRMRR agree well, but as shown, when using a less sensitive detector (such as MCT), the TRPL signal falls beneath the noise floor before the low-injection condition is satisfied, preventing an accurate lifetime extraction

The lifetime data from TMR, our µ-TRMRR technique, and TRPL (with MCT and InSb detectors) are compared in Fig. 6. We observe that the TMR, µ-TRMRR, and TRPL (with an InSb detector) all give similar lifetimes and temperature dependence, indicating that the increased sensitivity of the µ-TRMRR approach does not come with an associated penalty in the accuracy of lifetime extraction. However, the MCT detector TRPL data show a clear discrepancy in extracted lifetimes, particularly as temperature increases and emission from the material decreases in efficiency. This effect again points to the primary deficiency of the TRPL method as described earlier in the discussion of Fig. 2c, where weak emission and/or low detector sensitivity prevents the accurate measurement of low carrier injection lifetimes in the tail of the decay from poorly emitting samples. The use of an InSb detector yields bulk InAsSb lifetimes that closely match with our single-pixel µ-TRMRR, as the InSb detector is far more sensitive than the MCT detector, allowing for a more accurate fit further into the tail of the TRPL signal (low-injection carrier concentrations are now above the noise floor). However, the sensitivity comes with a significant speed and spectral range penalty (see Methods); furthermore, the sensitivity would still not be sufficient to time resolve a single, micro-scale pixel.

In summary, we have presented the μ-TRMRR technique, capable of measuring minority carrier lifetimes in micro-scaled volumes of optoelectronic materials without the need for contacts or current collection. When compared with the results from TRPL measurements on the same material using an MCT detector, we show that the μ-TRMRR technique achieves a comparable signal using a factor of >10^5^ less absorbed energy, which allows us to characterize micron-scaled volumes of weakly emitting IR materials. Our µ-TRMRR results match well with lifetimes extracted from both conventional TMR and TRPL measurements, pointing to the accuracy of our technique for measuring carrier lifetimes in weakly emitting or long-lifetime materials. As our technique measures the material response at microwave frequencies, light emission from our samples is not required and thus there are no collecting optics and optical detectors that have to be adjusted to cater to optoelectronic materials operating in different wavelengths. These advantages are especially important for the characterization of micron-scaled volumes, such as the InAsSb pixels studied here or potential future studies of nano-scaled volumes of 2D materials.

## Methods

### Fabrication

The material investigated in this work is grown by Molecular Beam Epitaxy and consists of a 1 μm-thick UID InAsSb layer grown above a 250 nm-thick AlAsSb sacrificial layer, with both layers’ lattice matched to the GaSb substrate. The background carrier concentration of the n-InAsSb was measured to be 2.11 × 10^16^ cm^−3^ and 2.4 × 10^17^ cm^−3^ at 77 K and 300 K, respectively, by Hall measurements, although this approach may overestimate the true carrier concentration^21^. Fabrication of the 24 μm × 24 μm InAsSb pixels and their transfer to carrier substrates are described in detail in ref. ^22^. The pixel period pre-transfer was ~30 μm, and following transfer to the thermal release tape the pixel array consisted of a >4 mm × 4 mm pattern with a fill factor of ~0.5. The SRRs are fabricated on a 500 µm-thick 99.6% aluminum oxide substrate via standard photolithography and metallization with Ti/Au layers of 10/500 nm on both the top (patterned) and bottom (continuous groundplane) of the substrate. The microstrip and SRR widths are both 50 μm. The SRR side length is 1 mm, with a 30 μm coupling gap between the SRR and microstrip busline. Individual pixels and hBN spacers are transferred to the 10 μm gap of the SRR using the pick-and-place process detailed in ref. ^23^.

### Optical characterization

The as-grown InAsSb and the transferred pixel array are both characterized by PL spectroscopy and TRPL. For the former, the samples are placed in an optical cryostat and optically pumped by a modulated 980 nm laser diode. Emission spectra are collected using amplitude modulation step-scan with a Bruker v80V Fourier transform IR spectrometer. In the TRPL experiment, samples in an optical microscope cryostat are excited by a ~1 ns pulsed 1064 nm laser emitting 6.8 µJ pulses at a 10 kHz repetition rate. Emitted light is collected by an off-axis parabolic mirror and focused onto a Kolmar LN_2_-cooled high-speed (~4 ns time constant) MCT detector using a germanium lens. The resulting MCT signal is recorded with a high-speed oscilloscope (with 50,000 averages, to increase signal to noise and allow for measurement of the tail of the response). In addition, we performed a second set of TRPL measurements on the as-grown InAsSb using a lower speed (~23 ns), higher responsivity InSb detector, and a 1535 nm laser (with ~5 ns pulses). Pulse energy in the TRPL experiments is controlled by the use of neutral density filters. The carrier lifetimes are extracted by a single exponential fit to the tail of the TRPL response, ideally corresponding to the low-injection regime of significant import for detector materials. Both PL and TRPL data are collected for a range of sample temperatures for both the bulk as-grown material and a large-area pixel array.

### Microwave characterization

For the TMR measurements, a tunable optical parametric oscillator with 10 ns pulse width is used to excite the sample above the InAsSb band edge and the modulated reflection from a 94 GHz microwave Gunn diode is collected by a microwave detector. The transient TMR signal is then amplified using a wide-bandwidth preamplifier and averaged on a digital oscilloscope. Details of the TMR measurement system can be found in ref. ^6^. For μ-TRMRR, the SRR circuits are measured in an Advanced Research Systems RF cryo-probe stage with a ZnSe window for optical access. RF *S*_21_ spectra of the SRR circuits are collected using an Anritsu Shockline MS46122A Vector Network Analyzer with short-open-load-thru calibrations performed to move the reference plane to the probe tips. For the µ-TRMRR experiments, the pixels are optically excited using the above 1064 nm pulsed laser. Excitation pulse energy is controlled via neutral density filters up to ND5. Excitation pulse intensity is calculated by measuring the 2D beam profile of the laser at the position of the pixel. Time response of the pixel is measured by driving the SRR circuit on resonance using an RF source generator at fixed frequency. The transmitted RF signal is collected by a Pasternack 8013 zero-bias Schottky diode (with a capacitance of 470 pF) and fed into a digital oscilloscope, and the resulting time response is collected and averaged. The response of the circuit to CW excitation is obtained by modulating a 785 nm laser, focused onto the pixel, at ~48 Hz. The circuit is driven on resonance and the circuit response is collected by the zero-bias Schottky diode and fed into a lock-in amplifier.

### Modeling and calculations

We analytically model our circuit in Mathematica using the lumped element model shown in Fig. 3b. Transmission line impedances and permittivities are calculated using standard microstrip expressions^24^. Reflection coefficients and input impedances are determined at each impedance mismatch and are calculated as a function of frequency along with the lumped element impedance of the SRR^25^. Using a commercial finite element method (FEM) simulation software (www.ansys.com/products/electronics/ansys-hfss), the surface currents and electric fields of our circuit were plotted. On resonance, we observe a divergence in the current on the busline, which is out of phase with the electric field across the coupling region, suggesting that our SRR is capacitively coupled to the microstrip busline. Thus, we model the resonator as a capacitively coupled shunt resistor-inductor-capacitor (RLC) circuit^26,27^.

To calculate excess carrier concentration, $\Delta n$, for pulsed excitation, the energy incident upon the pixel is determined by measuring the power and 2D spatial profile of the laser beam at the position of the sample. The pulse energy is simply the measured power divided by the repetition rate. As the beam width is larger than the pixel, the total energy incident upon the pixel is simply the product of the amplitude of the Gaussian fit to the beam profile, the pixel area, the cryostat window transmission, and the transmission at the InAsSb/air interface, resulting in the 35.5 fJ incident upon the single pixel in Fig. 5b; for 5c, as the pixel cluster is larger than the spot size, the total beam energy is multiplied by an approximate pixel array fill factor, as well as the window and interface transmissions, which results in ~16.6 nJ. For the CW excitation, we measure the beam power and 2D profile in a similar manner and then calculate an EHP generation rate (*G*) assuming all photons entering the pixel are absorbed. The carrier concentration will then be $\Delta n,\Delta p \propto G\tau (\Delta n,\Delta p)$, assuming a carrier lifetime $\tau (\Delta n,\Delta p)$. We obtain an approximate $\Delta n$ by taking the product of the calculated generation rate and a constant carrier lifetime ($\tau \sim 250$ns).

## Data Availability

Data that support these findings are available from the corresponding author upon request.
